# Microbial Community Succession Sustains Fish Diversity in the Upper Yangtze River Reserve

**DOI:** 10.1002/advs.202505928

**Published:** 2025-10-15

**Authors:** Jiaxin Huang, Xiaohan Dong, Xinxin Zhou, Zongqiang Qi, Ziwei Wang, Jiali Ran, Kaiyue Xiao, Xingyu Pan, Hong Chen, Zhihao Liu, Qiliang Chen, Huajun Yang, Yanjun Shen

**Affiliations:** ^1^ Laboratory of Water Ecological Health and Environmental Safety School of Life Sciences Chongqing Normal University Chongqing 401331 China; ^2^ Chongqing Key Laboratory of Conservation and Utilization of Freshwater Fishes Chongqing 401331 China; ^3^ Animal Biology Key Laboratory of Chongqing Education Commission Chongqing China; ^4^ Chongqing Rare and Endemic Fish National Nature Reserve Management Office Chongqing China

**Keywords:** community assembly mechanisms, environmental DNA, fish diversity, microbial community, multitrophic association network

## Abstract

Fish diversity in rivers is critical for aquatic ecosystem sustainability, with multitrophic microbial communities (bacteria, fungi, phytoplankton, zooplankton) playing key roles in energy transfer. This study in the upper Yangtze River's Fish National Nature Reserve (FNNR) used environmental DNA (eDNA) to investigate microbial succession and its relationship with fish diversity. Bacteria showed the highest alpha‐diversity, while zooplankton has the lowest beta‐diversity. Geographic location and total nitrogen emerged as primary drivers of microbial community succession. Bacteria and phytoplankton demonstrated stronger environmental adaptability but lower community turnover compared to fungi and zooplankton. Null model analysis revealed homogenizing processes dominated bacterial and fungal assembly, whereas heterogeneous processes shaped phytoplankton and zooplankton communities. Microbial association networks indicated distinct community structures in different river systems. Path modeling showed that multitrophic microbial communities negatively impacted fish diversity, but cross‐trophic interactions among microorganisms has positive effects. These findings highlight how microbial diversity supports fish communities and provide conservation insights by linking microbial processes to ecosystem health. The study emphasizes the importance of understanding microbial dynamics for adaptive management strategies in biodiversity preservation.

## Graphical abstract

Critical for river ecosystem sustainability, fish diversity relates to multitrophic microbes. This upper Yangtze FNNR study used eDNA to examine microbial succession‐fish links, finding key drivers, distinct assembly processes, and that microbial cross‐trophic interactions benefit fish, offering conservation insights.

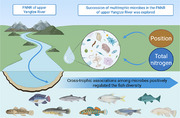

## 1 Introduction

Freshwater ecosystems are under threat from a number of factors, including overfishing, pollution, damming, and climate change, which can lead to biodiversity loss and ecosystem degradation.^[^ ^1^ ^]^ Fish are integral to maintaining ecological health and human well‐being within river ecosystems, being at the top of the food chain.^[^ ^2^ ^]^ Fish play an integral role in maintaining the balance of aquatic ecosystems by acting as both predators and prey, influencing nutrient cycling, and affecting the structure of river habitats.^[^ ^3^ ^]^ Furthermore, fish support significant recreational and cultural activities, including sport fishing and traditional practices, contributing to local economies and community well‐being.^[^ ^4^ ^]^ The Yangtze River Basin is recognized as the primary reservoir of freshwater fishery genetic resources in China.^[^ ^5^ ^]^ Its status as a geographical intersection of the Qinghai Tibet Plateau, the Yunnan‐Guizhou Plateau, and the Sichuan Basin results in a complex water environment and geological landscape, which in turn contributes to the biodiversity of fish genetic resources in the upper reaches of the Yangtze River.^[^ ^6^ ^]^ The effective protection of the integrity, authenticity, and biodiversity of this region can provide a solid foundation for sustainable fisheries development, biological evolution theory, and aquatic ecology research.^[^ ^7^ ^]^ Thus, a Fish National Nature Reserve (FNNR) has been established in the upper reaches of the Yangtze River to protect the rare and endemic fish diversity and enhance ecosystem resilience.^[^ ^8^ ^]^ Nevertheless, the levels of fish diversity in this region remain a cause for concern, underscoring the urgent need for further scientific strategies to ensure its effective maintenance.

Microbial communities, comprising both prokaryotic and eukaryotic organisms, play pivotal roles in preserving fish diversity within river ecosystems.^[^ ^6^ ^]^ For instance, bacteria are indispensable for the decomposition of organic matter and the recycling of nutrients, thereby supporting primary producers such as phytoplankton and enhancing habitat quality for fish.^[^ ^9^ ^]^ Furthermore, fungi, by decomposing complex organic materials such as submerged wood and leaf litter, contribute to the maintenance of the structural integrity of aquatic habitats, which are critical for fish spawning and shelter.^[^ ^10^ ^]^ Phytoplankton, in their capacity as primary producers, are pivotal in the conversion of sunlight into energy, thereby establishing the foundation of the aquatic food web.^[^ ^11^ ^]^ Furthermore, the consumption of phytoplankton by zooplankton ensures the availability of nutrition, thus maintaining the population of fish larvae and small fish species.^[^ ^12^ ^]^ The maintenance of diverse fish populations is contingent on the preservation of habitat quality and the availability of nutrients, both of which are ensured by the activity and diversity of microbes.^[^ ^13^ ^]^ In addition to the direct effects of microbial interactions, these interactions can also affect fish communities through the cascading effects of food webs.^[^ ^14^ ^]^ Diverse microbial interactions create stable ecosystems by ensuring nutrient recycling and energy flow through the food web.^[^ ^15^ ^]^ Disruptions to these interactions, such as pollution, habitat alteration, and climate change, can destabilize ecosystems and reduce fish diversity.^[^ ^16^ ^]^ Despite the evident association between microorganisms and fish diversity, the underlying mechanism of its deep internal correlation remains to be elucidated.

It is evident that microbial communities demonstrate intricate responses to environmental shifts, exerting an influence on ecological functions through alterations in alpha‐ and beta‐diversity, adaptation mechanisms, and community assembly processes.^[^ ^17^ ^]^ Alpha‐diversity, which denotes the diversity within a designated habitat or community, is subject to variation across different biomes, exhibiting distinct levels of microbial richness.^[^ ^18^ ^]^ Harsh environmental conditions can lead to extinctions, reducing alpha‐diversity, particularly for rare microbial species that are more sensitive to disturbances than common species.^[^ ^19^ ^]^ Beta‐diversity measures the variation in community composition between habitats or across spatial gradients.^[^ ^20^ ^]^ Intermediate switching rates may maximize beta diversity by allowing weaker species to coexist with stronger competitors.^[^ ^21^ ^]^ The ability of microbial communities to adapt to their environment involves a range of mechanisms that allow microbes to survive and thrive under varying conditions.^[^ ^22^ ^]^ Several indices have been explored to evaluate the environmental adaptation of plankton communities, including niche breadth and phylogenetic signal.^[^ ^23^ ^]^ Community assembly processes determine how species colonize and persist in an environment, which can be assigned to deterministic and stochastic processes.^[^ ^24^ ^]^ Deterministic assembly alters community composition and increases beta‐diversity by environmental filtering or competitive interactions.^[^ ^25^ ^]^ Conversely, stochastic processes, such as random birth and death events (termed ecological drift), chance dispersal of organisms, and random fluctuations in species abundances over time, also influence community structure.^[^ ^26^ ^]^ Typically, the responses of microbial communities belonging to different taxonomies or trophic to the same environmental conditions are very different.^[^ ^5^, ^27^ ^]^ It is evident that further exploration is required in order to gain a more comprehensive understanding of the intricate ecological succession processes and their relationships with regard to fish diversity.

This study obtained a series of water samples from the Fish National Nature Reserve (FNNR) in the upper Yangtze River and investigated the microbial communities across multiple trophic levels using eDNA‐based high‐throughput sequencing. The diversity and assembly mechanisms of these microbial communities were analyzed, and the association network among these microbial communities was constructed. The diversity of fish communities in the FNNR was also obtained by the eDNA technology. The study hypothesized that environmental conditions and multitrophic microbial communities could contribute to maintaining fish diversity in the FNNR, and the mechanisms underlying this relationship were explored. It is crucial to understand this information for the development of effective strategies to protect the unique ecosystem and its biodiversity.

## 2 Experimental Section

### 2.1 Study Area and Sample Collection

The FNNR of the upper Yangtze River is located within four provinces: Yunnan, Sichuan, Guizhou, and Chongqing. It encompasses the mainstream of the Yangtze River, the main tributaries of the Chishui River, as well as five tributaries of the Yangtze River: the Minjiang River, the Nanguang River, the Changning River, the Tuojiang River, and the Yongning River. The FNNR is located between the longitudes of 104°9′ E and 106°30′ E, and the latitudes of 27°29′ N and 29°4′ N. The total length of the river within the FNNR is 1138.31 km. The region is home to a diverse array of ichthyofauna, with 189 documented species, including 66 endemic to the upper Yangtze River reach and 21 classified as protected under national or local legislation. These species represent a significant component of China's biological heritage, holding substantial scientific, economic, and biodiversity value.

**Figure 1:**
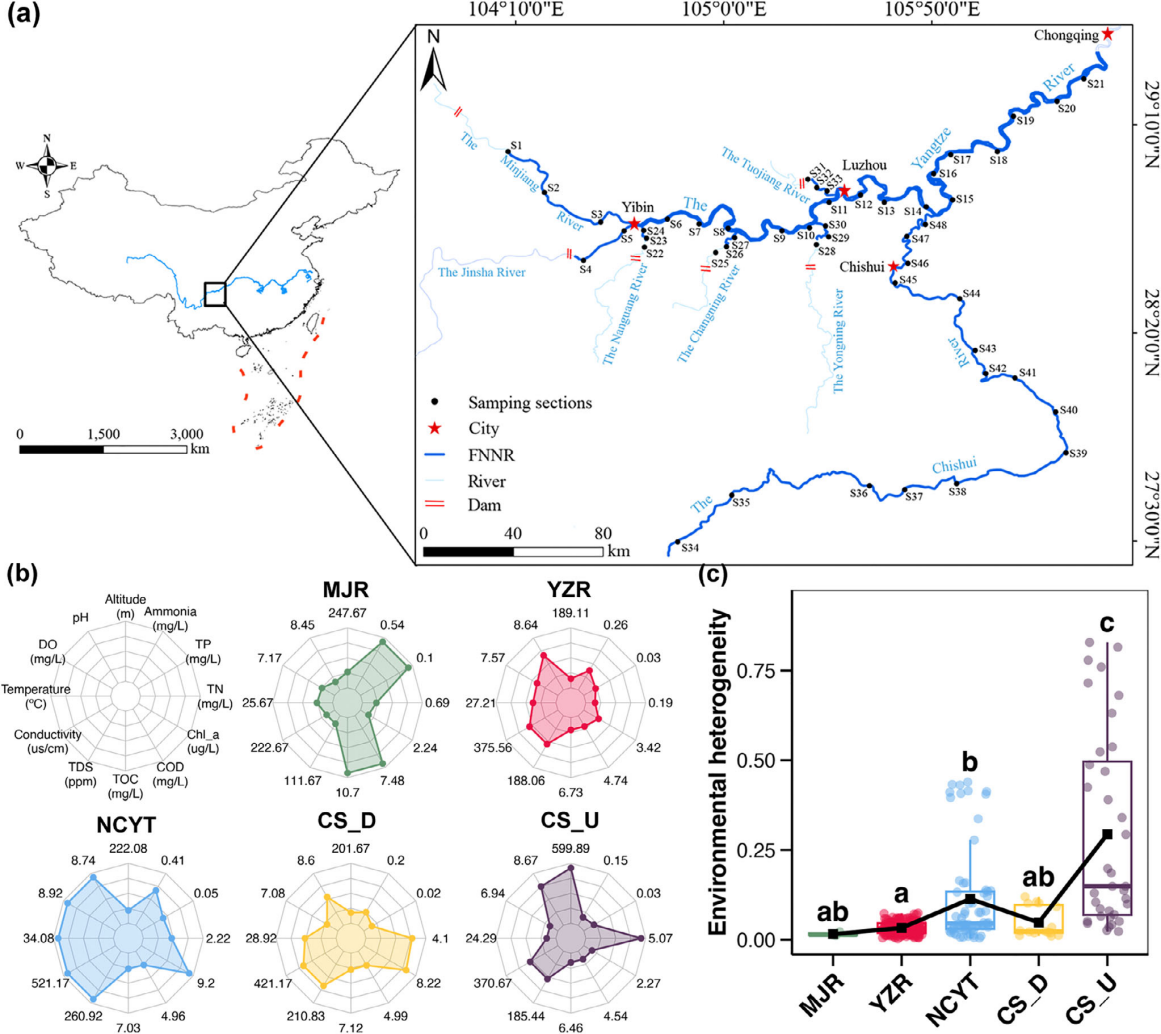
a) The map of sampling; b) Radder plots showing the differences in environmental conditions among different regions, with the points in each plot representing the average value of the corresponding factor among all samples belonging to the same region; c) Differences in the environmental heterogeneity among different regions, with the black square in each box representing the average value among all samples belonging to the same region. The presence of different lowercase letters above boxes indicates significant differences between regions (Tukey's HSD test, *p* <0.05).

The present study was conducted in August 2024. According to the geographical position, the FNNR was partitioned into 16 distinct river sections. Sampling points were strategically set up at the upper, middle, and lower reaches of each distinct river section (**Figure** **1**a). A 2.5 L water sampler (Wuhan Shuitiandi Instruments, Wuhan, China) was utilized to collect 3 L of water samples at each station. In total, 48 water samples were gathered. Immediately after collection in the field, these samples were placed in a refrigerated environment. Prior to every sampling event, all equipment was thoroughly disinfected with a 10% bleach solution and then rinsed with double‐distilled water. To further minimize the risk of contamination, disposable equipment was used for each sampling.^[^ ^28^ ^]^ In the laboratory, water samples from the same river section were combined to create a composite sample. This composite sample was then divided into three technical replicates, with each replicate containing precisely 2 L of the mixed water. The remaining 3 L of the composite sample were discarded.^[^ ^29^, ^30^, ^31^ ^]^ Subsequently, 1 L of the mixed water sample was filtered through 0.22 µm polycarbonate membranes (Millipore Corporation, Billerica, MA, USA). In cases where a large amount of sediment was present in the water samples, sterile medical gauze was used to pre‐filter the samples during the collection process.^[^ ^31^ ^]^ Before filtering each individual sample, the filtration equipment was disinfected to guarantee the absence of cross‐contamination. During the sample processing stage, 2 L of distilled water was used as a negative control to check for any exogenous DNA contamination. The filter membranes were then quick‐frozen in liquid nitrogen and stored at −80 °C until DNA extraction. Additional water samples (1 L) were temporarily stored at 4 °C for sequential environmental variable analyses. Prior to processing environmental DNA, performing PCR amplification, and conducting sequencing, it was essential to first carry out corresponding experiments on all negative controls. Only when the PCR reaction results of the negative controls were negative can sample processing proceed. The samples were grouped geographically, resulting in five distinct clusters: the Minjing River (MJR, S1–S3), the main stream of the Yangtze River (YZR, S4–S21), the Nanguang‐Changning‐Yongning‐Tuojiang River (NCYT, S22–S33), the upper reaches of the Chishui River (CS_U, S34–S42), and the lower reaches of the Chishui River (CS_D, S43–S48) (Figure 1a).

### 2.2 Measurements of Environmental Factors

Concurrent with sample collection, comprehensive environmental characterization was performed through multiparameter field measurements (Table S1, Supporting Information). The longitude, latitude, and elevation were acquired using a GPS device. Hydrochemical parameters, including electrical conductivity (EC), total dissolved solids (TDS), chemical oxygen demand (COD), and total organic carbon (TOC), were quantified in situ using an LS310 multiparameter probe (Shenzhen Linshang Technology Co., Ltd., Shenzhen, China) (Hou et al., 2013). Dissolved oxygen (DO) and water temperature (T) were measured with a Hanan dissolved oxygen meter (HI98193), while pH was determined using a HEMA pH meter (PH828). Nutrient species analysis for total nitrogen (TN), total phosphorus (TP), ammonium nitrogen (NH_3_‐N), and chlorophyll‐a (Chl_a) was conducted using a TE‐700PLUS multi‐parameter water quality analyzer (TIANER, Tianjin, China).

### 2.3 DNA Extraction and High‐Throughput Sequencing

The total genomic DNA of the water samples was extracted from the filters using a DNeasy PowerWater Kit (QIAGEN, CA, USA) in accordance with the manufacturer's instructions. Agarose gel electrophoresis (1% concentration) was used to ascertain the efficacy of the DNA extraction process. The quality of the DNA was measured using a NanoDrop ND‐1000 Spectrophotometer (NanoDrop, USA). The V3‐V4 regions of bacterial 16S rRNA gene, the ITS1 region of fungal ITS gene, the V9 region of eukaryotic 18S rRNA gene, and the 12S rRNA gene of fish were amplified by the primers of 341F‐806R (341F: 5′‐CCTAYGGGRBGCASCAG‐3′; 806R: 5′‐GGACTACNNGGGTATCTAAT‐3′;^[^ ^32^ ^]^), ITS1F‐ITS2R (ITS1F: 5′‐CTTGGTCATTTAGAGGAAGTAA‐3′; ITS2R: 5′‐GCTGCGTTCTTCATCGATGC‐3′;^[^ ^33^ ^]^), 1380F‐1510R (1380F: 5′‐CCCTGCCHTTTGTACACAC‐3′; 1510R: 5′‐CCTTCYGCAGGTTCACCTAC‐3′;^[^ ^34^ ^]^), and Tele02_F‐Tele02_R (Tele02_F: 5′‐AAACTCGTGCCAGCCACC‐3′; Tele02_R: 5′‐GGGTATCTAATCCCAGTTTC‐3′;^[^ ^35^ ^]^), respectively.

The PCR reactions were performed using a 20 µL mixture comprising 4 µL of 5 × FastPfu Buffer, 2 µL of 2.5 mM dNTPs, 0.8 µL of each primer (5 µM), 0.4 µL of FastPfu Polymerase, and 10 ng of template DNA. The detailed PCR processes were as follows: 95 °C for 2 min, followed by 25 cycles at 95 °C for 30 s, 55 °C for 30 s, and 72 °C for 30 s, and a final extension at 72 °C for 5 min. The amplicons were then extracted from 2% agarose gels and purified using the AxyPrep DNA Gel Extraction Kit (Axygen Biosciences, Union City, CA, U.S.) in accordance with the manufacturer's instructions. The quantity of the purified PCR products was then measured using Qubit3.0 (Life Invitrogen), and every twenty‐four amplicons with different barcodes were then mixed equally. The DNA was then fragmented using an ultrasonic instrument. The DNA library was then obtained by unwinding the DNA duplex product from PCR amplification and disrupting the uncircularized DNA molecules. These libraries were finally sequenced using a next‐generation sequencing platform (MGI‐G99) (Shanghai BIOZERON Biotech. Co., Ltd) with PE300 mode according to the standard protocols.

### 2.4 Data Processing

To annotate multitrophic microbial communities, the raw data of 16S rRNA, ITS, and 18S rRNA genes in the fastq format were subjected to a preliminary demultiplexing procedure. This was performed using in‐house Perl scripts, with the demultiplexing process being guided by the barcode sequence information for each individual sample. Quality control of the sequenced reads was performed based on the following standards: i) average Phred scores higher than 20, ii) no ambiguous bases, iii) homopolymer runs lower than 8, iv) no mismatches in the primers, and v) read length longer than 250 bp.^[^ ^36^ ^]^ Subsequently, the DADA2 plugin unit in the QIIME2 program was utilized to assemble the paired reads, eliminate the chimera, and cluster clean data into amplicon sequence variants (ASVs).^[^ ^37^ ^]^ The ASVs of bacterial 16S rRNA genes and fungal ITS genes were then classified according to taxonomy, with the SILVA (Release 138)^[^ ^38^ ^]^ and UNITE^[^ ^39^ ^]^ databases serving as reference sources, respectively. For the ASVs of eukaryotic 18S rRNA gene, they were also annotated by the SILVA (Release 138) database, and then treated by an in‐house R script to separate phytoplankton and zooplankton.^[^ ^40^ ^]^ A randomly selected subset of sequences (48720 for bacteria, 52350 for fungi, 49482 for phytoplankton, and 47333 for zooplankton, respectively) from each sample was utilized to standardize the sequencing effort across samples, and the ASV abundance tables for different microbial communities were obtained, respectively.

For fish species annotation, raw sequencing reads of 12S rRNA gene were quality‐filtered using Trimmomatic v0.36 with the following parameters^[^ ^41^ ^]^: 1) removal of 3′‐end bases with Phred scores <20 via a sliding window approach (10 bp window size; window quality cutoff = 20), 2) discarding trimmed reads shorter than 100 bp. Paired‐end reads were merged into contiguous sequences using FLASH v1.2.11 with a minimum overlap length of 10 bp and a maximum mismatch density threshold of 0.2 to eliminate suboptimal assemblies.^[^ ^42^ ^]^ Demultiplexing was performed through exact matching of barcode and primer sequences (zero mismatch allowed), followed by orientation standardization. Chimeric sequences were detected and removed through hybrid reference‐based (GOLD database v11.0) and de novo approaches using USEARCH v10 (UPARSE algorithm). High‐quality sequences were clustered into operational taxonomic units (OTUs) at 97% similarity threshold via the UPARSE‐OTU algorithm. Taxonomic annotation was conducted through BLASTn searches (uclust algorithm, identity ≥99%, E‐value ≤1e^−5^) against two reference databases: 1) MitoFish (Release 2023.01) for fish‐specific classification, and 2) NCBI nt database (2024_04 release) with subsequent exclusion of non‐piscine sequences.^[^ ^43^ ^]^ OTUs with read counts <10 were systematically filtered prior to downstream analyses.

### 2.5 Statistics Analysis

All statistical analyses were performed in the R v4.4.2 platform^[^ ^44^ ^]^ and visualized by the “ggplot2” package.^[^ ^45^ ^]^ The Chao1 index and Bray‐Curtis distance were calculated using the “vegan” package^[^ ^46^ ^]^ to represent the alpha‐ and beta‐diversity of microbial communities and fish communities, respectively. The differences in alpha‐ and beta‐diversity among different communities or geographic regions were then subjected to a Tukey's HSD test, utilizing the “multcomp” package.^[^ ^47^ ^]^ Principal coordinate analysis (PCoA) and the adonis test based on the Bray‐Curtis distance were further performed using the “ape” packages^[^ ^48^ ^]^ to evaluate the variations in microbial communities among different geographic regions.

The dissimilarity among samples based on environmental variables was calculated on the basis of the Euclidean distance, with the “vegan” package being utilized for this purpose. The heterogeneity of environmental conditions for each pair of samples within a single geographic region was obtained using a previously reported method.^[^ ^49^ ^]^ Furthermore, the differences in environmental heterogeneity among different regions were examined by means of Tukey's HSD test. The geographic distance between each of the two sampling stations was calculated by the “geosphere” package^[^ ^50^ ^]^ based on the coordinates of latitude and longitude. To estimate the relative importance of environmental and geographic effects on the microbial communities, we performed the distance‐decay of community similarity (DDCS) between the alpha‐ and beta‐diversity of microbial communities and the environmental dissimilarity or geographic distance, respectively, by linear regression. In addition, Mantel tests were also executed by the “LinkET” package^[^ ^51^ ^]^ to determine the correlations between environmental or geographic variables with the alpha‐ and beta‐diversity of microbial communities.

To explore the underlying mechanisms of the variations of community composition structure, we decomposed the beta‐diversity of microbial communities to species replacement (Repl) and richness difference (RichDiff) using the “agricolae” and “adespatial” packages.^[^ ^52^ ^]^ In addition, the niche breadth of each ASV was calculated based on Levin's niche breadth index using the “MicroNiche” package.^[^ ^53^ ^]^ Then, the habitat niche breadth of each sample was defined as the average niche breadth of ASVs detected in the corresponding sample.^[^ ^54^ ^]^ The differences in niche breadth and habitat niche breadth among different microbial communities were measured by Tukey's HSD test to evaluate their environmental adaptation. The phylogenetic signals of different microbial groups to each environmental variable were further appraised by Blomberg's K statistic (“picante” package).^[^ ^55^ ^]^ A value of Blomberg's K statistic closer to 0 represents a convergent pattern of evolution, and a value higher than 1 implies stronger phylogenetic signals for environmental change. Moreover, to examine community assembly mechanisms, we applied the entire‐community null model analysis based on the beta nearest taxon index (betaNTI) and the Raup‐Crick metric (RC).^[^ ^56^ ^]^ The betaNTI can be used to distinguish between deterministic (|betaNTI| >2) and stochastic (|betaNTI| ≤2) processes. Deterministic processes can then be further categorized into two distinct types: homogeneous selection (betaNTI <−2) and heterogeneous selection (betaNTI >2). The stochastic processes can be further categorized into three events: homogeneous dispersal (RC <−0.95), dispersal limitation (RC >0.95), and drift (|RC| ≤0.95).^[^ ^57^ ^]^

The microbial association network of microbial communities was constructed on the basis of Spearman rank correlations between the relative abundance of each microbial ASV. ASVs detected in more than 60% of samples and with an average relative abundance higher than 0.1% were selected for correlation analysis. The identification of association events was based on statistically robust correlations (|correlation coefficient| >0.8 with Benjamini‐Hochberg adjusted *p*‐value <0.05).^[^ ^58^ ^]^ A total of 10 types of potential microbial associations were divided into four categories: positive within‐trophic associations (WTAs), negative WTAs, positive cross‐trophic associations (CTAs), and negative CTAs. Subsequently, subnetworks for each sample were extracted, and the topological parameters of these networks were obtained using the “igraph” package.^[^ ^59^ ^]^ These topological parameters included the number of nodes and edges, average degree, density, average clustering coefficient, modularity, and number of different associations. The differences in these topological parameters among different regions were then tested using Tukey's HSD test.

The present study employed random forest models, utilizing the “randomForest” package^[^ ^60^ ^]^ to identify the predictors of the alpha‐ and beta‐diversity of fish communities based on environmental and geographic factors, alpha‐ and beta‐diversity of microbial communities, community assembly process of microbial communities, and the topological parameters of microbial association networks. Furthermore, partial least squares path modeling (PLS‐PM) was performed using the “plspm” package^[^ ^61^ ^]^ to evaluate the direct and indirect effects of the aforementioned factors on the alpha‐ and beta‐diversity of fish communities. Finally, the gamma‐diversity of fish communities in different geographic regions was calculated by the “specpool” function in the “vegan” package. The Rao quadratic entropy was performed to assess the contribution of alpha‐ and beta‐diversity to the gamma‐diversity of the fish community.^[^ ^62^ ^]^

## 3 Results

### 3.1 Variations in Environmental Conditions

A comparison was made of the differences in environmental conditions among the various regions of the FNNR (Figure 1b; Figure S1, Supporting Information). The results indicated that the nutritional level was highest in MJR (Figure 1b), which exhibited significantly higher levels of ammonia, TP, COD, and TOC compared to other regions (Tukey's HSD test, *p* <0.05, Figure S1, Supporting Information). The NCYT regions exhibited the highest levels of water temperature, pH, DO, Chl_a, conductivity, and TDS (Figure 1b), and among them, water temperature, Chl_a, conductivity, and TDS were found to be significantly higher than those in some other regions (Tukey's HSD test, *p* <0.05, Figure S1, Supporting Information). The CS_U region was distinguished by its significantly higher altitude and TN content compared to other regions (Tukey's HSD test, *p* <0.05, Figure S1, Supporting Information). In contrast, the environmental conditions of YZR were relatively moderate in all aspects (Figure 1b). Furthermore, the comparisons of environmental heterogeneity indicated that the CS_U region exhibited the maximum fluctuations of environmental conditions, followed by the NCYT region (Figure 1c). Conversely, the environmental conditions in other regions exhibited relative stability (Figure 1c).

### 3.2 Alpha‐ and Beta‐Diversity of Multitrophic Microbial Communities

**Figure 2:**
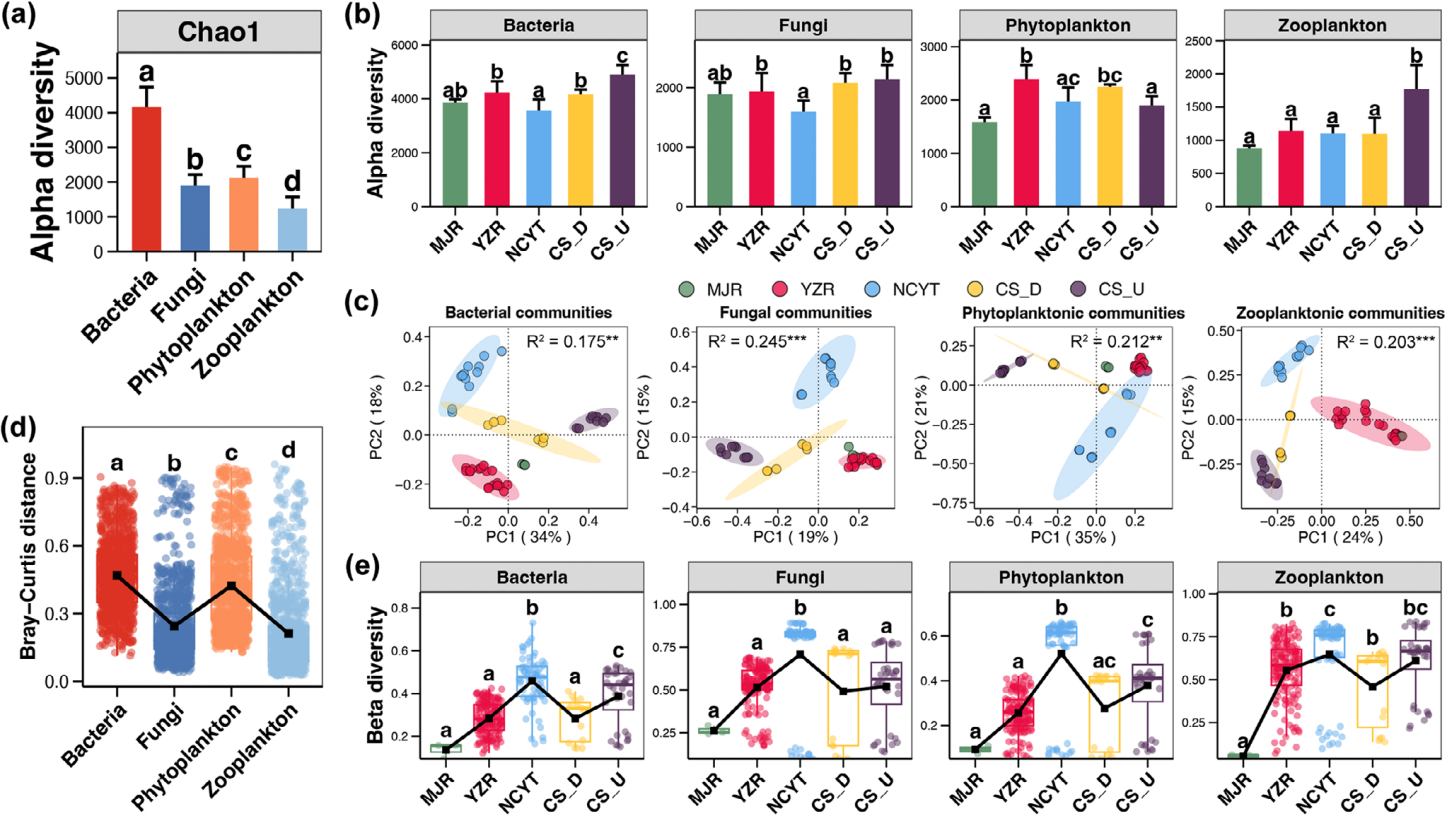
a) Discrepancies in the alpha‐diversity of disparate microbial communities. b) Discrepancies in the alpha‐diversity of individual microbial communities among divergent regions. The height of the bar denotes the mean value of alpha‐diversity of each microbial community among all samples (a) or samples from the same region (b), with the error bar denoting the standard deviation. c) Principal coordinates analysis (PCoA) and Adonis test revealing the cluster distribution of microbial communities among different regions. d) Differences in the beta‐diversity distance of different microbial communities. e) Differences in the beta‐diversity distance of a single microbial community among different regions. The black square in each box represents the average value among all samples belonging to the same region. Different lowercase letters above boxes of the same sub‐figure represent significant differences between different microbial communities or regions (Tukey's HSD test, *p* <0.05).

In the FNNR, the alpha‐diversity of the bacterial community was found to be highest, followed by that of the phytoplankton and fungi, and the lowest value was observed in the zooplankton (Tukey's HSD test, *p* <0.05, **Figure** **2**a). In each region of the FNNR, the rank of alpha‐diversity among the different microbial communities was found to be almost identical to that in all samples (Figure S2, Supporting Information). The only exception to this was in MJR, where the alpha‐diversity of the fungal community was higher than that of the phytoplankton (Figure S2, Supporting Information). Focusing on the microbial community at a single community level, the lowest alpha‐diversity of bacteria and fungi was observed in the NCYT region, while it was highest in the CS_U region (Tukey's HSD test, *p* <0.05, Figure 2b). Conversely, the alpha‐diversity of phytoplankton and zooplankton was observed in the MJR region, while it was highest in the YZR and CS_U regions, respectively (Figure 2b).

PCoA plots demonstrated the separation of the clusters of all four microbial communities across different regions. Geographic regions were found to have a significant effect on the variations of microbial communities (Adonis test, *p* <0.05, Figure 2c). The communities from the NCYT and CS_U regions were distinctly separated from those in the YZR, while communities in the MJR and CS_D regions exhibited closer proximity to the YZR samples, particularly in the case of MJR (Figure 2c). The analysis of beta‐diversity among disparate microbial communities further substantiated this finding, with bacteria in the FNNR exhibiting the most pronounced variations, followed by phytoplankton and fungi, and zooplankton demonstrating the most stability (Tukey's HSD test, *p* <0.05, Figure 2d). In contrast to the alpha‐diversity, a statistically significant variation in the rank of beta‐diversity was observed among the microbial communities of individual regions and the entire FNNR (Tukey's HSD test, *p* <0.05, Figure S3, Supporting Information). The beta‐diversity of fungi and zooplankton was found to be significantly higher than that of bacteria and phytoplankton in most regions, with the exception of MJR, where zooplankton exhibited the highest levels (Tukey's HSD test, *p* <0.05, Figure S3, Supporting Information). In the MJR region, the diversity of fungi was the highest, while the diversity of zooplankton was the lowest among all microbial communities (Figure S3, Supporting Information). It is noteworthy that the diversity of fungi was the highest in the NCYT region and the lowest in the MJR region for all four microbial communities (Figure 2e).

### 3.3 Associations Between Microbial Communities and Environmental or Geographic Factors

**Figure 3:**
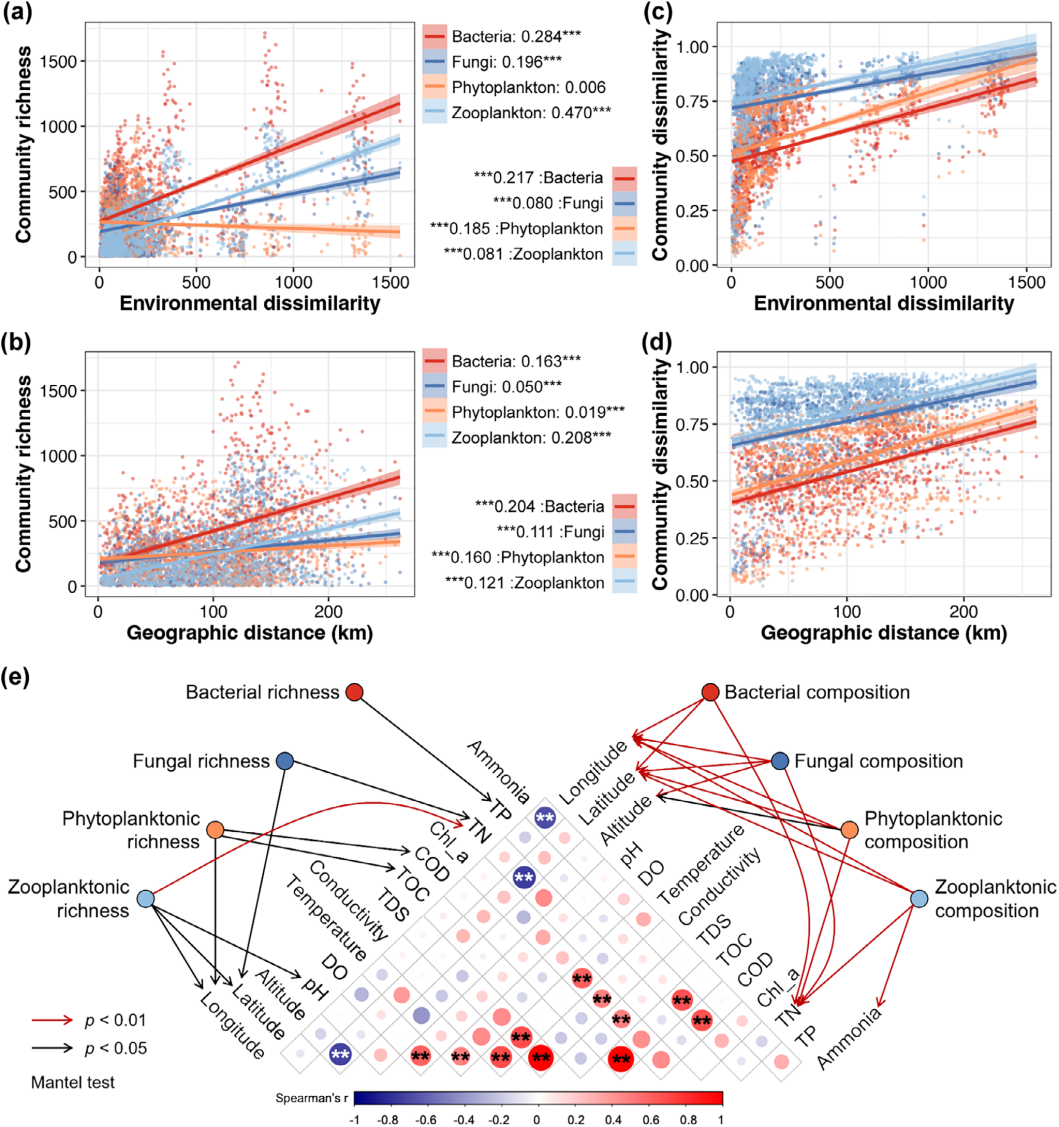
The distance‐decay of community similarity between the microbial communities is depicted against environmental dissimilarity (a, alpha‐diversity; c, beta‐diversity) and geographic distance (b, alpha‐diversity; d, beta‐diversity). The Mantel test correlations between the alpha‐ and beta‐diversity of microbial communities with environmental and geographic factors are demonstrated in (e). Asterisks indicate statistical significance (* *p* <0.05, ** *p* <0.01, *** *p* <0.001).

A significant degree of DDCS was obtained between the alpha‐diversity of microbial communities with both environmental and geographic conditions in the entire NNFR, with the exception of the alpha‐diversity of phytoplankton with environmental factors (linear regression, *p* <0.05, **Figure** **3**a,b). The strength of associations was strongest for zooplankton, followed by bacteria and fungi, and weakest for phytoplankton, with both environmental and geographic conditions (Figure 3a,b). In addition, the associations with environmental factors were much tighter than those with geographic distance for bacteria, fungi, and zooplankton (Figure 3a,b). Furthermore, a significant DDCS was identified between the beta‐diversity of all four microbial communities and both environmental and geographic conditions (linear regression, *p* <0.05, Figure 3c,d). The strength of associations was strongest for bacteria, followed by phytoplankton, and relatively weaker for zooplankton and fungi, with both environmental and geographic conditions (Figure 3c,d). The DDCS between bacteria and phytoplankton with environmental factors were found to be relatively close to those with geographic distance, while the associations for fungi and zooplankton were slightly tighter with geographic distance (Figure 3c,d).

Furthermore, the correlations between alpha‐ and beta‐diversity of microbial communities with single environmental and geographic factors were examined using the Mantel test. The alpha‐diversity of bacteria demonstrated a significant correlation with TP; the alpha‐diversity of fungi exhibited a significant correlation with TN and latitude; significant correlations were also identified between the alpha‐diversity of phytoplankton with COD, TOC, and longitude; and the alpha‐diversity of zooplankton exhibited a significant correlation with longitude, latitude, pH, and TN (*p* <0.05, Figure 3e). For beta‐diversity, longitude, latitude, and TN were observed to be significantly correlated to all four microbial communities (*p* <0.05, Figure 3e). Significant correlations were also obtained between altitude and the beta‐diversity of fungal and phytoplanktonic communities, as well as ammonia and the beta‐diversity of zooplankton (*p* <0.05, Figure 3e).

### 3.4 Community Assembly Mechanisms of Microbial Communities

**Figure 4:**
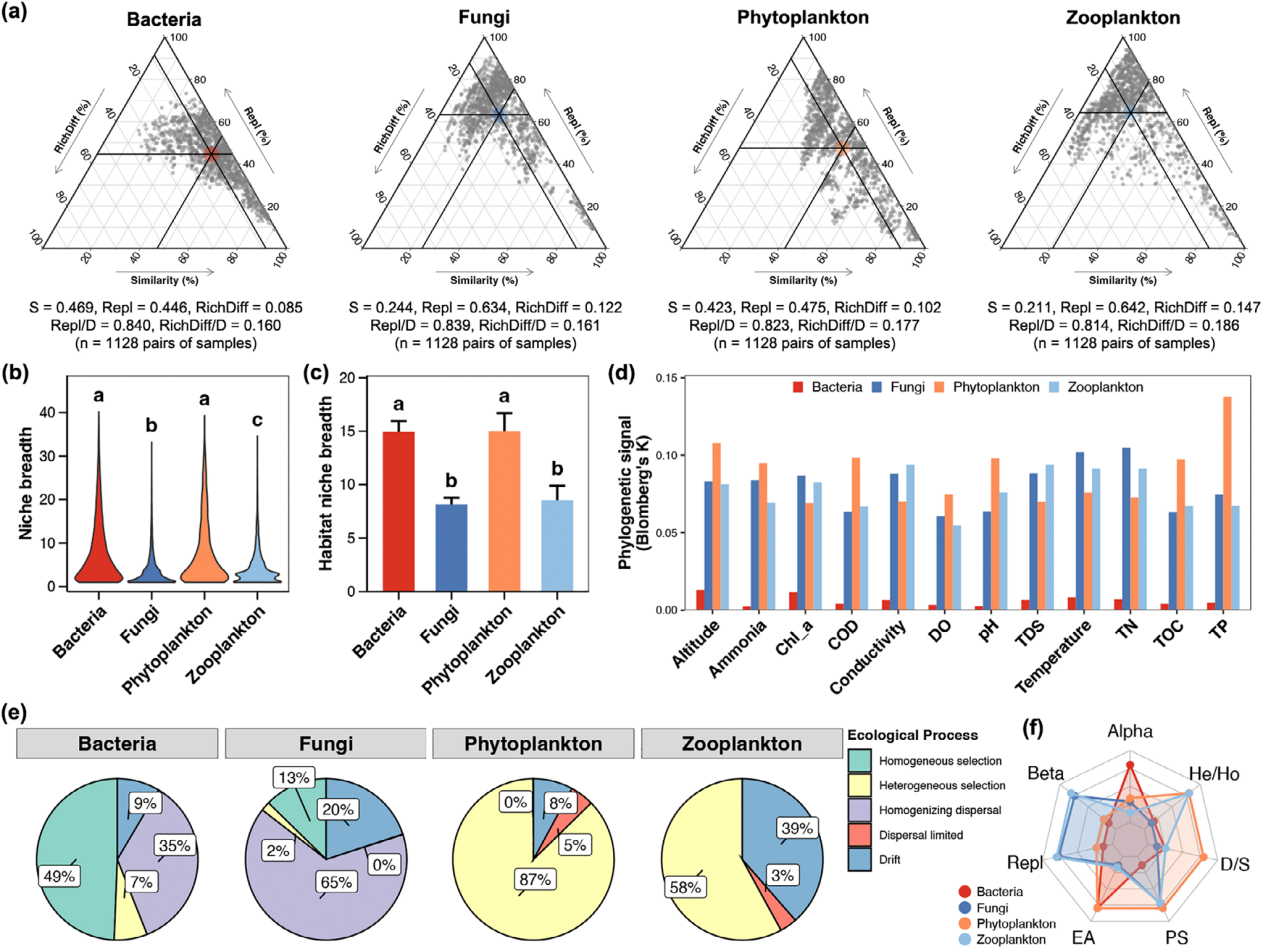
a) presents triangular plots illustrating the contribution of ecological processes (i.e., species replacement and richness difference) to the variations of microbial communities. Each data point represents a pair of samples. The position of each point is determined by a triplet of values from the similarity (S) = 1 – D (dissimilarity), Repl (species replacement), RichDiff (richness difference) matrices; it should be noted that each triplet sums to 1. The large point in each sub‐figure represents the central position of all points, and the black lines give the corresponding position in each axis (mean value of all points). b) Differences in the niche breadth of different microbial communities. c) Differences in the habitat niche breadth of different microbial communities. The height of the bar represents the average value of each microbial community among all samples, and the error bar represents the standard deviation. Different lowercase letters above boxes of the same sub‐figure represent significant differences between different microbial communities (Tukey's HSD test, *p* <0.05). d) Phylogenetic signal of different microbial communities for each environmental factor. e) The relative importance of different ecological processes for the assembly of microbial communities. f) Conceptual frameworks for the community assembly mechanisms among different microbial communities. The points represent the average value of the corresponding factor among all samples belonging to the same microbial community. Alpha, alpha‐diversity; Beta, beta‐diversity; Repl, species replacement; EA, environmental adaptation (niche breadth); PS, phylogenetic signal; D/S, ratio of deterministic and stochastic assembly; He/Ho: ratio of heterogeneous and homogenizing processes.

The beta‐diversity of microbial communities was initially decomposed into Repl and DiffRich, and the differences among different microbes were subsequently compared (**Figure** **4**a). The analysis revealed that Repl was a dominant factor influencing the beta‐diversity of all four microbial communities, with a contribution that was particularly pronounced in the case of fungi (0.634) and zooplankton (0.642), as compared to bacteria (0.446) and phytoplankton (0.475) (Figure 4a). Conversely, the bacterial and phytoplanktonic communities exhibited significantly higher niche breadth at both the ASV and habitat levels in comparison to the fungi and zooplankton (Tukey's HSD test, *p* <0.05, Figure 4b,c). Focusing on the phylogenetic signal, the bacterial community exhibited the lowest value for all environmental factors; the values for fungi and zooplankton were similar, with minor uncertain variations to different environmental factors; and the phytoplanktonic community possessed the highest phylogenetic signals in certain environmental factors, such as altitude, ammonia, COD, DO, pH, TOC, and TP (Figure 4d). The assembly of bacterial and fungal communities was governed by homogeneous selection and homogenizing dispersal, respectively (Figure 4e). In contrast, the assembly of phytoplankton and zooplankton was governed by heterogeneous selection (Figure 4e). Taken together, the bacterial community exhibited the highest alpha‐diversity but the lowest phylogenetic signal, while the fungal and zooplanktonic communities demonstrated the highest beta‐diversity and the most species replacement; Bacteria and phytoplankton exhibited greater adaptability to environmental changes, while phytoplankton and zooplankton were more influenced by the hetero‐assembly; and the phytoplanktonic community received the most selective assembly (Figure 4f).

### 3.5 Distinct Microbial Associations Among Different Regions

**Figure 5:**
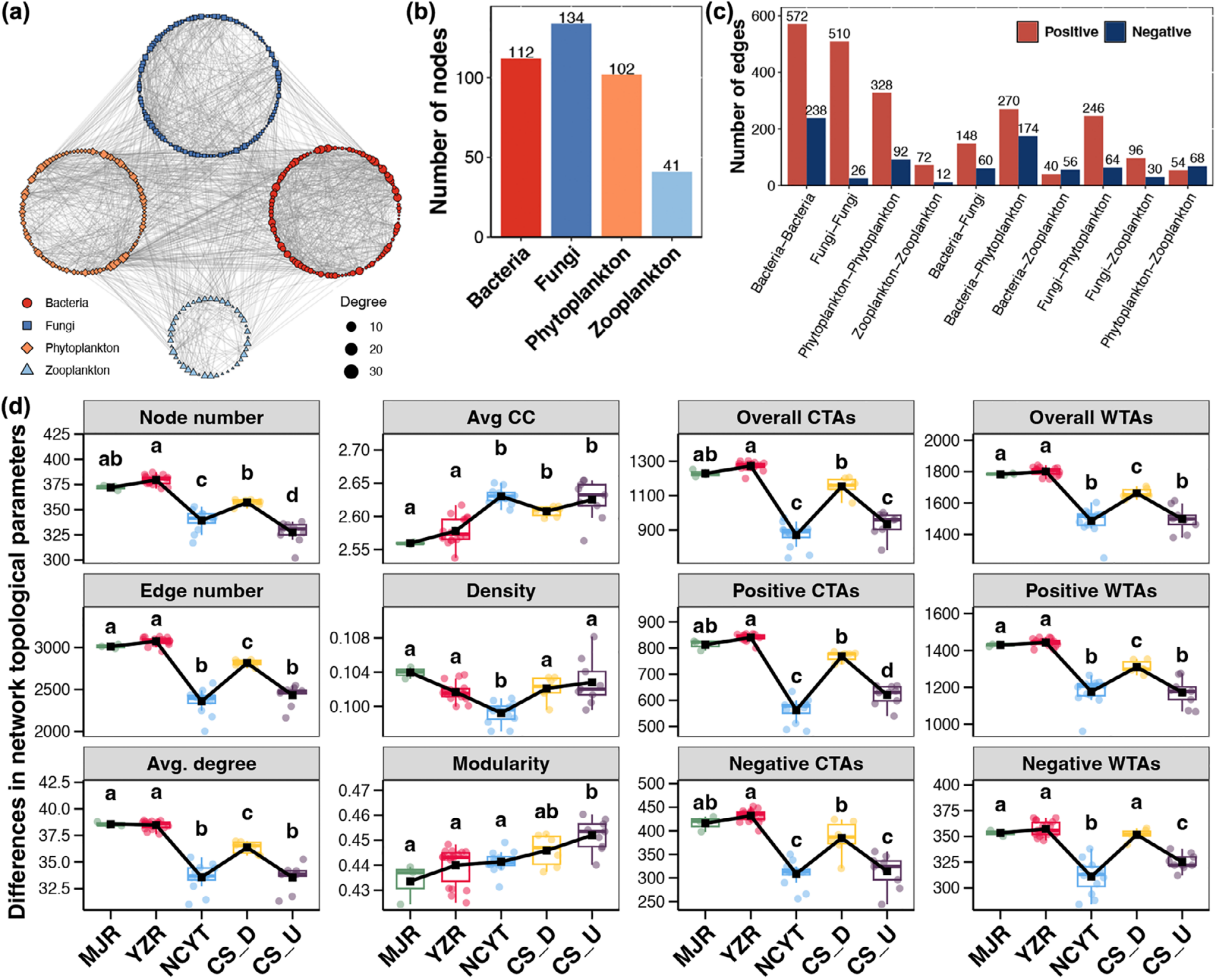
a) a visual representation of the microbial association network in the FNNR. b) The statistical analysis of node numbers across different microbial communities. c) The number of positive and negative edges among all WTAs and CTAs. d) The differences in the topological parameters of the microbial association network across different regions. The black square in each box represents the average value among all samples belonging to the same region. Different lowercase letters above boxes of the same sub‐figure represent significant differences between different regions (Tukey's HSD test, *p* <0.05). Avg. degree, average degree; Avg. CC, average clustering coefficient; CTAs, cross‐trophic associations; WTAs, within‐trophic associations.

The microbial association network of the FNNR has been constructed, comprising 112 bacterial ASVs, 134 fungal ASVs, 102 phytoplanktonic ASVs, and 41 zooplanktonic ASVs (**Figure** **5**a,b). The majority of associations in this network were positive, and among them, associations among bacteria, fungi, and phytoplankton occupied the majority, irrespective of WTAs or CTAs (Figure 5c). A further comparison of the topological parameters of the network among different regions was conducted, revealing that the majority of these parameters were elevated in MJR and YZR, followed by CS_D, and diminished in NCYT and CS_U regions. This encompassed the numbers of nodes, edges, CTAs, and WTAs, along with the average degree (Figure 5d). Conversely, the average clustering coefficient and modularity were lower in MJR and YZR compared to other regions (Figure 5d). These results indicated that the microbial interaction network in the MJR and YZR regions was more complex and integrated.

### 3.6 Variations in Fish Diversity

**Figure 6:**
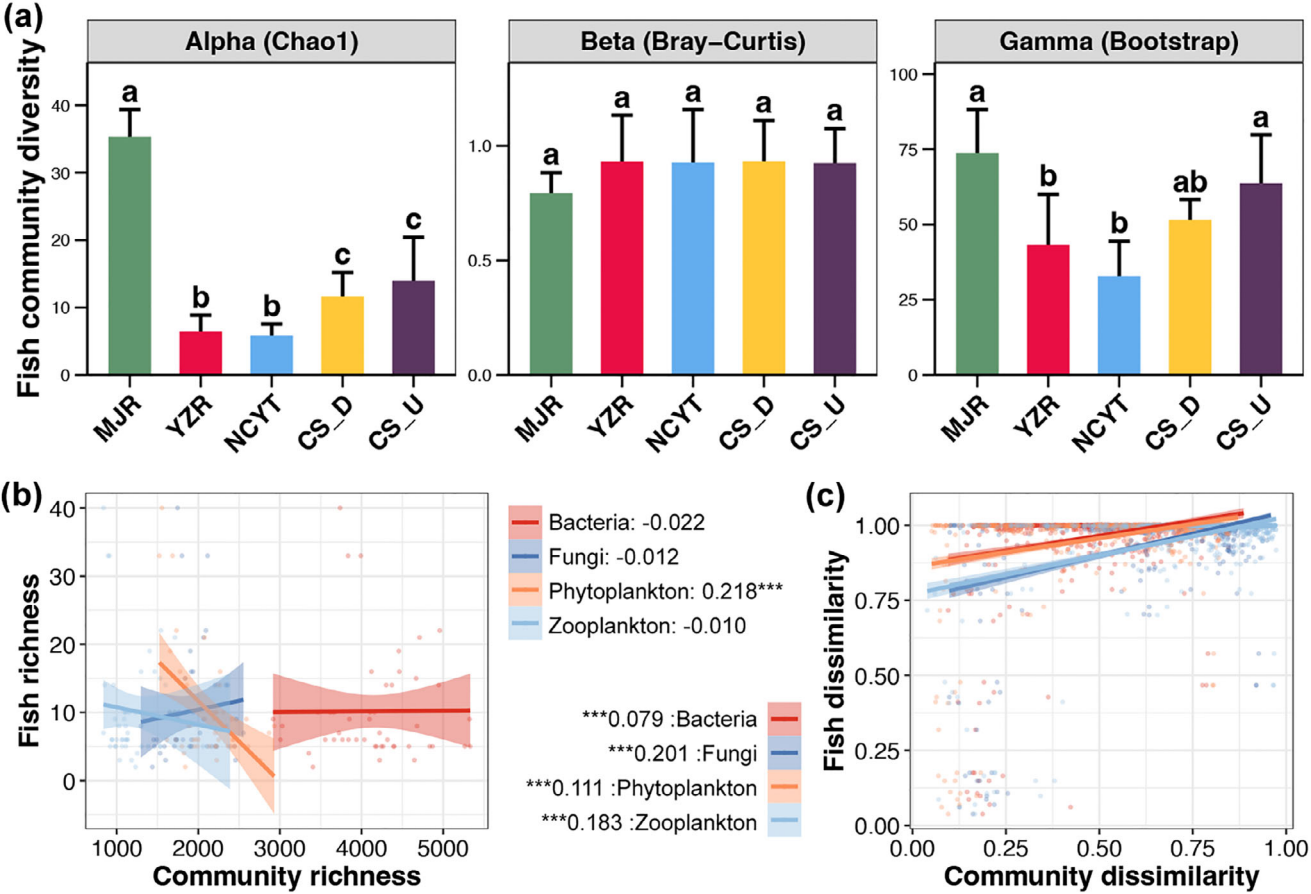
a) Differences in the alpha‐, beta‐, and gamma‐diversity of fish community among different regions. The height of the bar represents the average value of each diversity among all samples, and the error bar represents the standard deviation. Different lowercase letters above boxes of the same sub‐figure represent significant differences between different regions (Tukey's HSD test, *p* <0.05). Linear regression of the alpha‐ b) or beta‐diversity c) between the fish community and different microbial communities. Asterisks indicate statistical significance (* *p* <0.05, ** *p* <0.01, *** *p* <0.001).

The alpha‐diversity of the fish community in the MJR region was found to be significantly higher than in other regions in the FNNR. Furthermore, the alpha‐diversity of the CS river was found to be higher than that of the YZR and NCYT regions (Tukey's HSD test, *p* <0.05, **Figure** **6**a). Conversely, the beta‐diversity of fish communities did not demonstrate a clear variation among the regions (Tukey's HSD test, *p* >0.05, Figure 6a). Furthermore, the gamma‐diversity of fish communities was observed to be highest in the MJR and CS_U regions, and lowest in the NCYT region (Tukey's HSD test, *p* <0.05, Figure 6a). Subsequent analyses investigated the correlations between the alpha‐ and beta‐diversity of fish communities and the various microbial communities. The alpha‐diversity of phytoplankton exhibited a significant correlation with fish richness, albeit in a negative direction (linear regression, *p* <0.05, Figure 6b). In contrast, a significant correlation was observed between the beta‐diversity of fish communities and all four microbial communities (linear regression, *p* <0.05, Figure 6c). The correlation strength of beta‐diversity was more pronounced for fungi (0.201) and zooplankton (0.183), which exhibited higher R^2^ compared to bacteria (0.079) and phytoplankton (0.111) (Figure 6c).

### 3.7 Driving Factors for Maintaining Fish Diversity

**Figure 7:**
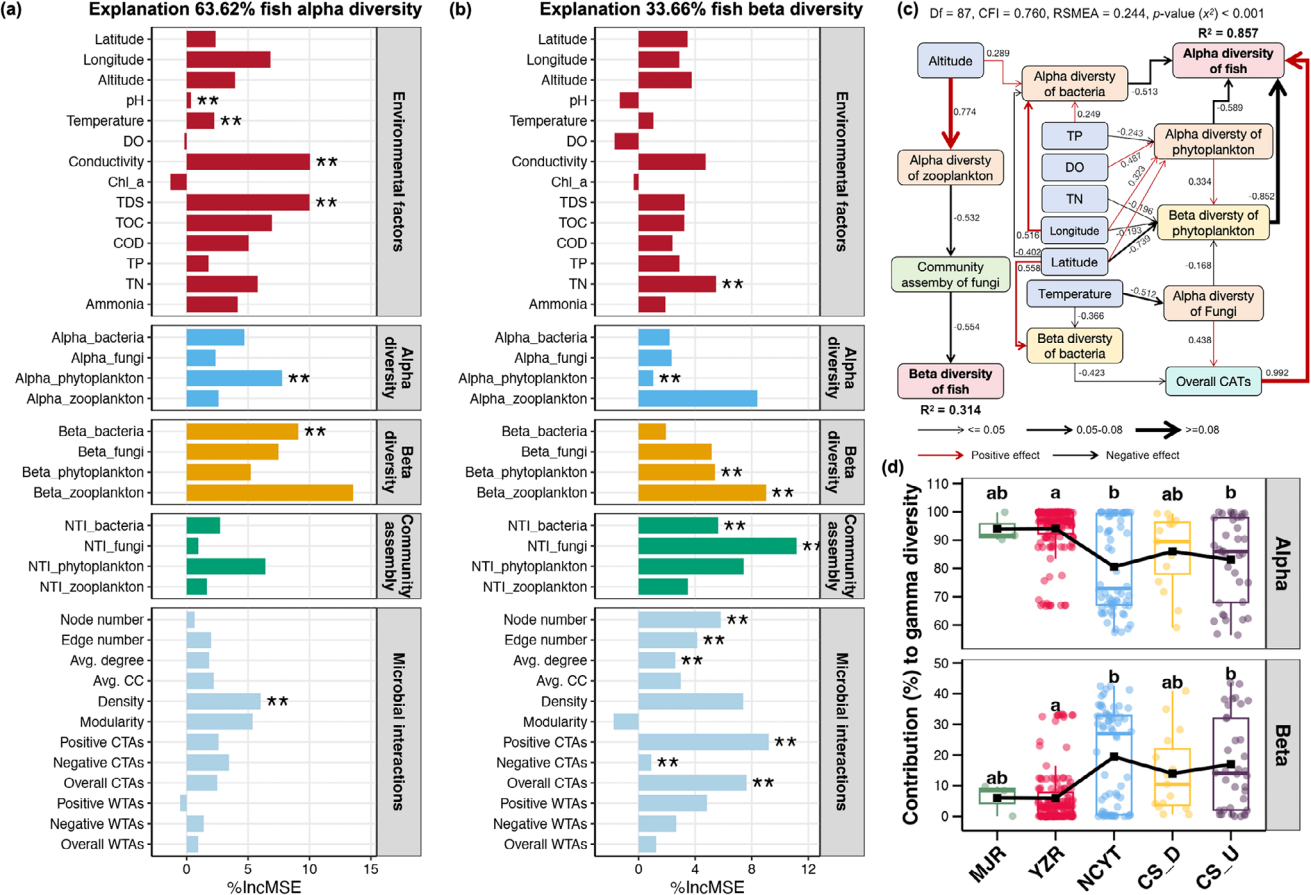
The results of the random forest model, which demonstrate the significance of environmental factors, alpha‐ and beta‐diversity, and assembly mechanisms of microbial communities, and microbial association networks for the alpha‐ a) and beta‐diversity b) of fish communities. Asterisks denote statistical significance (* *p* <0.05, ** *p* <0.01, *** *p* <0.001). c) A partial least squares path model (PLS‐PM), which demonstrates the direct and indirect effects of environmental factors and microbial communities on the alpha‐ and beta‐diversity of fish communities. It should be noted that only significant links are shown in the figure, with red and black arrows denoting positive and negative relationships, respectively. The path coefficients are shown adjacent to the arrows. d) a comparison of the contribution of alpha‐ and beta‐diversity to the gamma‐diversity of fish communities among different regions. The black square in each box is indicative of the average value among all samples belonging to the same region. Different lowercase letters above boxes of the same sub‐figure represent significant differences between different regions (Tukey's HSD test, *p* <0.05).

In order to predict variations in alpha‐ and beta‐diversity of the fish community, RF models were constructed based on environmental and microbial factors. The RF model explained 63.62% of the variations in the alpha‐diversity of the fish community (**Figure** **7**a). The RF model revealed significant correlations between the alpha‐diversity of the fish community and environmental factors, including pH, conductivity, TDS, alpha‐diversity of phytoplankton, beta‐diversity of bacteria, and microbial association network density (Figure 7a). In contrast, the RF model explained a comparatively lower proportion (33.66%) of variations in the beta‐diversity of the fish community (Figure 7b). The TN, alpha‐diversity of phytoplankton, beta‐diversity of phytoplankton and zooplankton, community assembly of bacteria and fungi, and several topological parameters of the microbial association network were significantly correlated to the beta‐diversity of the fish community (Figure 7b).

In the subsequent stage of the study, a PLS‐PM was constructed in order to explore the driving factors for maintaining the fish diversity (Figure 7c). The beta‐diversity of the fish community was found to be negatively regulated by the community assembly of fungi, which in turn was influenced by the alpha‐diversity of zooplankton and ultimately controlled by the altitude (Figure 7c). The regulation network for the alpha‐diversity of the fish community was found to be more complex. The alpha‐diversity of fish was observed to be negatively influenced by the alpha‐diversity of bacteria and phytoplankton, as well as the beta‐diversity of phytoplankton, with these effects being regulated by multiple environmental and geographic factors (Figure 7c). Of particular significance was the positive regulation of the alpha‐diversity of fish by the overall CATs in the microbial association network, a process further influenced by the alpha‐diversity of fungi and beta‐diversity of bacteria, and ultimately controlled by latitude and water temperature (Figure 7c). Finally, the contributions of alpha‐ and beta‐diversity to the gamma‐diversity of fish community were estimated. The alpha‐diversity was found to have a significant impact on the gamma‐diversity of fish, contributing more than 90% to the MJR and YZR regions (Figure 7d). However, in the NCYT and the CS river regions, this contribution was found to decrease significantly, reaching 80%‐85% (Tukey's HSD test, *p* <0.05, Figure 7d).

## 4 Discussion

In this study, the strength of DDCS of microbial communities was found to be more closely associated with environmental dissimilarity than with geographic distances, particularly in the context of alpha‐diversity (Figure 3). This phenomenon can be attributed to the stronger control exerted by environmental selection over microbial diversity in comparison to dispersal limitations. This phenomenon has been observed to occur on a large scale or across distinct biotas.^[^ ^63^ ^]^ The significant difference in environmental heterogeneity among different regions (Figure 1c) indicates that environmental disparities increase with the distance between each sampling station, causing the divergence of microbial diversity.^[^ ^64^ ^]^ These findings redefine microbial biogeography, emphasizing that “everything is everywhere, but the environment selects”.^[^ ^65^ ^]^ In contrast to the geographically dominant succession, microbial functions could be more predictable when tied to environmental filtering.^[^ ^66^ ^]^ Correlations between the single environmental or spatial factors to microbial diversity had also been investigated in the FNNR. However, geographic location (longitude and latitude) and TN content had been shown as the potential key drivers to regulate the alpha‐ and beta‐diversity of microbial communities (Figure 3e). This discrepancy may be attributable to the multifaceted metrics employed to calculate environmental dissimilarity in this study, while geographical factors are limited to longitude and latitude. It is challenging to predict changes in microbial communities in complex natural environments through a single environmental factor^[^ ^67^ ^]^ except under relatively extreme pressure conditions.^[^ ^68^ ^]^ Consequently, future research should integrate multi‐dimensional environmental data to predict microbial responses to anthropogenic disturbances.^[^ ^69^, ^70^ ^]^ Consequently, the preservation of microbial diversity necessitates the prioritization of environmental heterogeneity over the establishment of purely spatial conservation networks.^[^ ^64^ ^]^ Restoration initiatives must encompass the replication of pivotal environmental conditions, as opposed to the reliance on proximity to source communities.^[^ ^71^ ^]^

Results of this study indicated Repl as the dominant process contributing to the variations of beta‐diversity of all four different microbial groups (Figure 4a). Similar results were also found in these microbial groups from other ecosystems, such as bacterial and fungal communities in river‐lake continuum,^[^ ^72^ ^]^ phytoplankton in estuaries,^[^ ^11^ ^]^ and zooplankton in floodplains.^[^ ^73^ ^]^ These findings highlighted the universal importance of turnover processes in maintaining and structuring biodiversity in aquatic ecosystems. Nevertheless, the processes that underpin the assembly of microbial communities vary significantly among different microbial communities.^[^ ^74^ ^]^ This variation is attributable to differences in their biological traits, ecological niches, and environmental interactions. In this study, the DDCS of community dissimilarity was found to be strongest for bacteria, and second, powerful for community richness based on both environmental and spatial factors (Figure 3). The higher metabolic flexibility of bacterial communities leads to greater influence from selection, and their smaller size contributes to greater dispersal potential.^[^ ^75^ ^]^ These biological traits result in homogenizing processes (both homogeneous selection and homogenizing dispersal) dominating the assembly of bacterial communities in the FNNR (Figure 4e). A similar set of rules may also apply to the assembly of fungal communities, which exhibited only slightly weaker DDCS than bacterial communities.^[^ ^76^ ^]^ This is due to the somewhat larger size and more specialists in these communities.^[^ ^76^ ^]^ In contrast, the body size of phytoplankton and zooplankton is remarkably larger than that of bacteria and fungi.^[^ ^77^ ^]^ This feature is known to limit dispersal over large spatial scales, thus rendering local environmental conditions critical for community assembly.^[^ ^78^ ^]^ The results of this study demonstrate that heterogeneous selection has a significant impact on the assembly of phytoplankton and zooplankton in the FNNR (Figure 4e), thereby supporting the proposed perspective. A notable observation is that the richness of zooplankton exhibited the closest DDCS compared to other microbial communities (Figure 3). As the highest trophic level in microbial communities, geographic and environmental conditions impose greater restrictions on zooplankton than on other.^[^ ^79^ ^]^ On the other hand, the level of alpha‐diversity was lowest in zooplankton among all four microbial communities (Figure 2a), suggesting that changes of the same scale can lead to greater differences. In summary, the balance between selection and dispersal varies across microbial communities due to differences in size, metabolic traits, and alpha‐diversity levels. These differences made the assembly of phytoplankton and zooplankton more predictable under stable conditions, while the ecosystem functioning of bacteria and fungi was more unpredictable. But it should be noted that all samples in the present study were collected from a single‐time sampling. Relying on single‐time (snapshot) sampling to infer community assembly processes can substantially bias conclusions by failing to capture temporal dynamics, misrepresenting species interactions, and conflating stochastic and deterministic drivers.^[^ ^56^, ^57^ ^]^ Further repeated sampling across relevant temporal scales could be essential to obtain robust, mechanistic insights into how communities assemble and change over time.

Despite the relatively low level of alpha‐diversity, zooplankton have been identified as valuable indicators of river ecological condition due to their sensitivity to environmental changes and their role in the aquatic food web.^[^ ^80^ ^]^ A microbial association network was constructed in this study, and in it, the associations between zooplankton and other microbial trophies were clearly lower (Figure 5). Rivers are dynamic systems characterized by fast‐flowing water, which limits the establishment of stable microbial association networks.^[^ ^81^ ^]^ In such conditions, only microorganisms with rapid growth and high renewal rates can thrive, thereby reducing the likelihood of sustained interactions between zooplankton and other microorganisms.^[^ ^82^ ^]^ Furthermore, the diet of zooplankton in rivers consists primarily of low‐trophic microorganisms, with no evidence of symbiotic or mutualistic relationships.^[^ ^83^ ^]^ Instead, interactions are predominantly predatory or opportunistic in nature, such as grazing on algae or bacteria when available.^[^ ^84^ ^]^ This phenomenon has the potential to result in less efficient nutrient cycling in the FNNR, due to the impact of ecosystem productivity without cross‐trophic interactions.^[^ ^85^ ^]^ The reliance of zooplankton on environmental factors rather than microbial partnerships also renders them more sensitive to disturbances such as pollution or climate change, which can destabilize riverine food webs.^[^ ^86^ ^]^ Consequently, the analysis of zooplankton communities can offer valuable insights into the health and sustainability of river ecosystems. It is therefore recommended that greater attention be paid to the monitoring of zooplankton diversity in the FNNR.

Furthermore, the microbial association networks in the NCYT and Chishui River have been shown to be less complex and fragmented in comparison to the MJR and YZR regions (Figure 5d). The presence of complex microbial networks has been demonstrated to facilitate more efficient and diverse biogeochemical cycling activities, thereby influencing the overall functioning of river ecosystems.^[^ ^87^ ^]^ Furthermore, complex networks can provide a buffer against environmental disturbances by allowing microbes to adapt and respond more effectively to changes with greater resilience.^[^ ^88^ ^]^ In contrast, fragmented microbial networks can impair essential ecosystem services, including water purification and nutrient cycling, which can have cascading effects on aquatic life.^[^ ^13^ ^]^ Furthermore, the long‐term degradation of aquatic ecosystems is generally indicated by fragmented microbial communities.^[^ ^89^ ^]^ Consequently, more targeted strategies need to be developed for the NCYT and Chishui River basin within the FNNR. For instance, a restoration and protection zone could be established in the NCYT region, with priority given to restoring the biological association networks in this river section to enhance the ecosystem functioning and resilience provided by microbial communities.

The primary objective of this study is to investigate the maintenance mechanisms of fish diversity within the FNNR. The findings indicate that environmental conditions and microbial communities exert a substantial influence on the alpha‐diversity of the fish community, while their impact on the beta‐diversity of the fish community is comparatively negligible (Figure 7). The impact of environmental factors on local habitat conditions is direct, influencing the species that can survive and thrive in a given area, thereby shaping alpha‐diversity of fish.^[^ ^90^ ^]^ Microbial communities also play a role in shaping alpha‐diversity by influencing ecosystem processes such as nutrient cycling, which affects habitat quality and creates strong deterministic filtering at the local level.^[^ ^91^ ^]^ Conversely, while environmental gradients can influence the beta‐diversity of fish communities, this influence can be mitigated by dispersal‐driven similarity.^[^ ^92^ ^]^ Additionally, beta‐diversity can be driven by species replacement or richness difference, which are less sensitive to local environmental factors and more influenced by regional dynamics such as geographic barriers or dispersal constraints.^[^ ^93^ ^]^ The findings of this study provide further evidence in support of previous research (Figure 7c), with the dominant indirect effects of altitude on the beta‐diversity of fish communities partly corroborating the conclusions of the earlier study. Since alpha‐diversity of fish is closely linked to local environmental conditions and microbial communities, it can be deduced that fish communities are likely to be highly sensitive to habitat degradation or pollution.^[^ ^90^ ^]^ While the comprehensive composition of fish communities was found to be only minimally impacted, disturbances were found to disproportionately affect local species richness.^[^ ^94^ ^]^ Given that multiple rare and endemic fish survive in the studied FNNR, the loss of unique local assemblages due to reduced regional biodiversity will cause irreparable ecological damage. The findings of this study highlight the need for integrated conservation approaches that consider both local habitat protection and regional connectivity to sustain biodiversity at multiple scales.

As indicated by previous studies, microbial communities interact with fish in complex ways that have the potential to be both beneficial and detrimental, depending on the context and environmental conditions.^[^ ^95^, ^96^ ^]^ Generally, a positive correlation between microbial diversity and fish community should be expected, due to the support provided by microbes for nutrient cycling and food webs.^[^ ^11^ ^]^ However, the findings of this study demonstrate predominantly adverse impacts of microorganisms on fish diversity, particularly the negative effects of bacteria and phytoplankton on the alpha‐diversity of fish communities (Figure 7). The observed negative correlations can be attributed to several interconnected ecological and environmental factors. First, elevated nutrient loads, especially phosphorus, frequently result in eutrophication, which can stimulate bacterial and phytoplankton growth but also generate stressful conditions for fish by modifying water quality.^[^ ^97^, ^98^ ^]^ Secondly, although bacteria and phytoplankton occupy different ecological niches, they are also indirectly connected through nutrient cycling.^[^ ^99^ ^]^ High microbial activity has been shown to deplete resources, thereby creating unfavorable conditions for fish.^[^ ^43^ ^]^ Furthermore, increased microbial diversity has been observed in downstream sections of rivers due to nutrient accumulation, but reduced fish diversity due to habitat degradation or fragmentation.^[^ ^100^ ^]^ Moreover, both bacteria and phytoplankton as the primary productivity, and most fish do not directly absorb energy from them.^[^ ^101^ ^]^ In contrast, fish nutrition comes more from the middle trophic level, such as zooplankton, which transfer energy to the upper trophic level by feeding on bacteria and phytoplankton.^[^ ^102^ ^]^ Thus, a greater number of fish require more mid‐trophic level organisms, which in turn leads to the massive consumption of primary trophic levels through trophic cascades.^[^ ^103^ ^]^ This might be reflected in the negative correlation between fish and the diversity of bacteria or phytoplankton. However, it should be noted that reducing microbial diversity does not necessarily guarantee the maintenance of fish diversity in the FNNR. It is crucial to recognize that microbial communities are the foundation of ecosystem functions and services. Consequently, any alteration to these communities, whether intentional or not, may result in ecosystem collapse and more severe catastrophic consequences.^[^ ^17^ ^]^

It is interesting to note that a positive effect was observed between the associations among distinct trophic microorganisms and the alpha‐diversity of fish in the FNNR (Figure 7). This is supported by the findings of Trombetta et al.^[^ ^104^ ^]^ who demonstrated that CTAs among microorganisms improve energy transfer efficiency within the microbial food web, ultimately benefiting fish populations. Meanwhile, CTAs contribute to ecosystem stability by maintaining functional redundancy and supporting fish habitat sustainability.^[^ ^105^ ^]^ Furthermore, a healthy microbial association network indirectly benefits fish by ensuring a steady supply of food resources^[^ ^106^ ^]^ and providing shelter and breeding grounds.^[^ ^107^ ^]^ By implementing reasonable strategies, it is possible to enhance these associations and ensure the long‐term sustainability of aquatic biodiversity. For instance, controlled nutrient enrichment has been demonstrated to result in a substantial increase in CTAs, thereby promoting ecosystem functionality.^[^ ^108^ ^]^ Furthermore, restoring natural river flow patterns has been shown to ensure the continuity of microbial interactions across spatial scales, thereby benefiting fish diversity.^[^ ^109^ ^]^ Furthermore, the mitigation of anthropogenic stressors, such as pollution, sedimentation, and damming, can contribute to the preservation of the stability of microbial association networks.^[^ ^110^ ^]^ In conclusion, the intricate relationship between environmental microbial communities and fish diversity necessitates a comprehensive consideration of the analysis results from diverse perspectives to formulate a rational strategy, thereby averting the potential regret arising from hasty decisions.

## 5 Conclusion

This study has yielded a more profound comprehension of the succession of multitrophic microbial communities in the FNNR of the upper Yangtze River. The diversity exhibited marked distinctions among the various microbial communities, with the highest levels observed in bacteria and the lowest in zooplankton at both the alpha and beta levels. Furthermore, variations in the alpha‐ and beta‐diversity of a single community across different geographic regions were also identified. Significant DDCS with microbial communities was observed between geographic and environmental factors. Geographic location and TN content were identified as key factors regulating the variations of microbial communities. Distinct assembly mechanisms were uncovered among different microbial communities, with stronger environmental adaptability for bacteria and phytoplankton and higher species replacement for fungi and zooplankton. Furthermore, a more complex and integrated microbial association network was identified in the MJR and YZR compared to other regions. In addition, variations in fish diversity across the FNNR were revealed, with higher values observed in the MJR and CS_U regions. The alpha diversity of fish was found to be more influenced by environmental conditions and microbial communities, which also contribute to the majority of the gamma‐diversity of fish. The maintenance of fish diversity in the FNNR is significantly influenced by associations among different trophic microorganisms, highlighting the crucial ecological ramifications of multitrophic microbial communities in river ecosystems, particularly in regard to fish diversity conservation. Future research endeavors should focus on exploring strategies that could enhance fish conservation and improve ecosystem health through the regulation of microbial communities.

## Conflict of Interest

The authors declare no conflict of interest.

## Author Contributions

J.H. performed writing the original draft, visualization, investigation, formal analysis, and data curation. X.D. performed investigation, formal analysis, and data curation. X.Z. performed data curation. J.R. K.X., X.P., and H.C. performed the investigation. Z.W., Z.Q., Z.L., Q.C., and H.Y. performed writing, reviewing, and editing. Y.S. performed writing, reviewing, editing, supervision, resources, investigation, and conceptualization.

## Supporting information



Supporting Information
