# Supplementary material for: Microbial Community Succession Sustains Fish Diversity in the Upper Yangtze River Reserve
Source: Adv Sci (Weinh). 2025 Oct 15;12(47):e05928. doi: 10.1002/advs.202505928 (PMC12713060; doi:10.1002/advs.202505928)
Supplement: Supplementary file 1 — Supporting Information [file ADVS-12-e05928-s001.docx]

**Supporting information for**

#### **Microbial community succession sustains fish diversity in the Upper Yangtze River Reserve**

Jiaxin Huang^1,2^, Xiaohan Dong^1,2^, Xinxin Zhou^1,2^, Zongqiang Qi^1,2^, Ziwei Wang^1,2^, Jiali Ran^1^, Kaiyue Xiao^1^, Xingyu Pan^1^, Hong Chen^1^, Zhihao Liu^1,2,3^, Qiliang Chen^1,2,3^, Huajun Yang^4^, Yanjun Shen^1,2,3,*^

^1^Laboratory of Water Ecological Health and Environmental Safety, School of Life Sciences, Chongqing Normal University, Chongqing 401331, China.

^2^Chongqing Key Laboratory of Conservation and Utilization of Freshwater Fishes, Chongqing 401331, China.

^3^Animal Biology Key Laboratory of Chongqing Education Commission, Chongqing, China.

^4^Chongqing Rare and Endemic Fish National Nature Reserve Management Office, Chongqing, China.

*Corresponding author, E-mail address: shenyanjun@cqnu.edu.cn (Yanjun Shen).


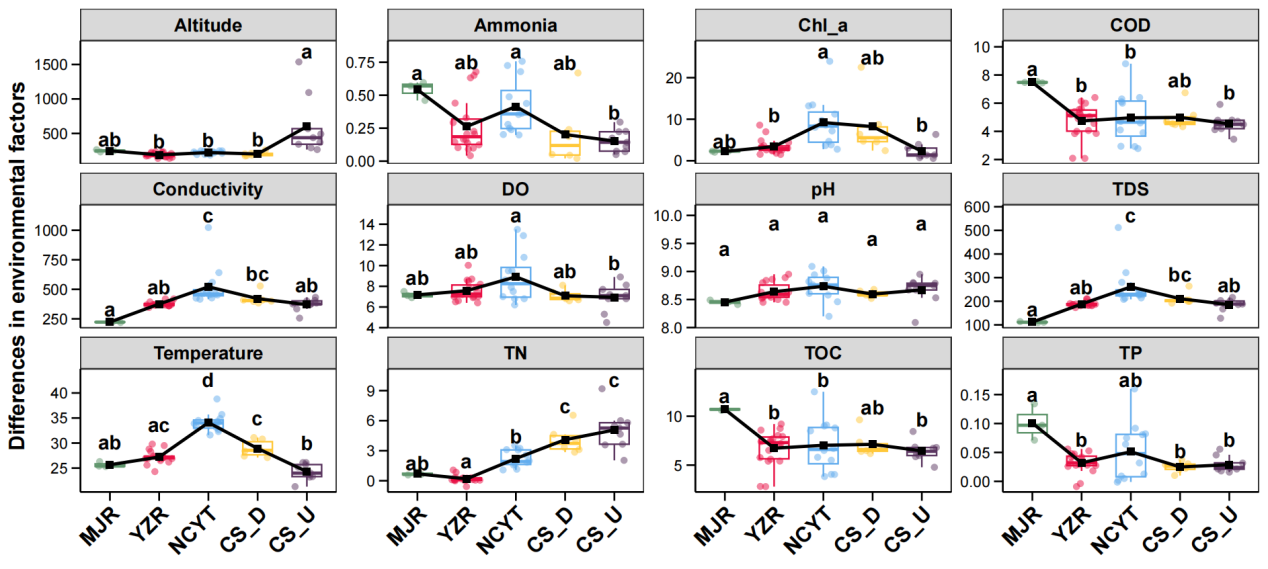


**Figure S1.** Differences in the environmental factors among different geographic regions. The black square in each box represents the average value among all samples belonged to the same region. Different lowercase letters above boxes of the same sub-figure represent significant differences between different regions (Tukey's HSD test, *p* <0.05).


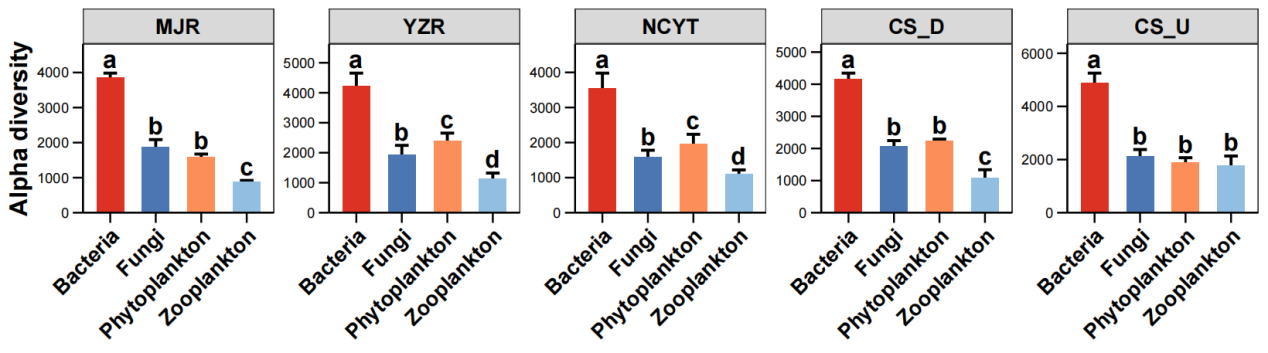


**Figure S2.** Differences in the alpha-diversity distance of different microbial metacommunities in a single geographic regions. he black square in each box represents the average value among all samples belonged to the same region. Different lowercase letters above boxes of the same sub-figure represent significant differences between different microbial metacommunities (Tukey's HSD test, *p* <0.05).


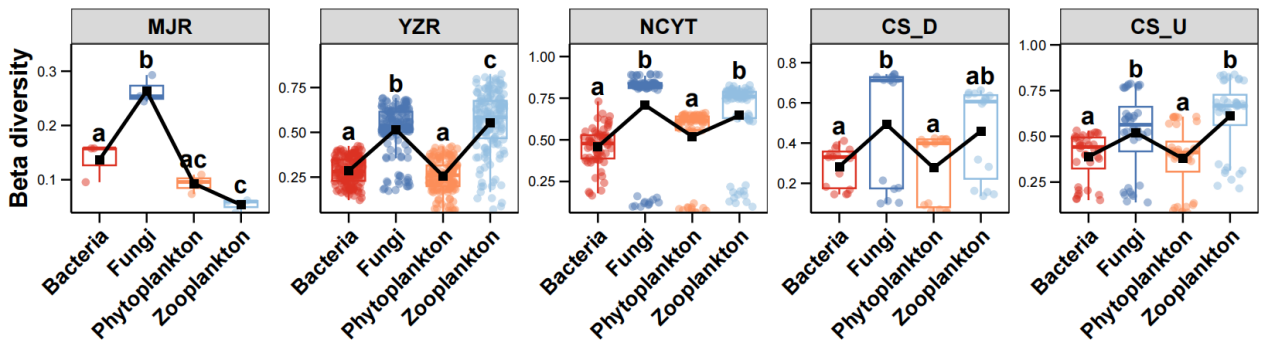


**Figure S3.** Differences in the beta-diversity distance of different microbial metacommunities in a single geographic regions. he black square in each box represents the average value among all samples belonged to the same region. Different lowercase letters above boxes of the same sub-figure represent significant differences between different microbial metacommunities (Tukey's HSD test, *p* <0.05).

**Table S1*.*** The environmental factor of the Fish National Nature Reserve (FNNR) in the upper Yangtze River.

| Sample site | Longitude  (°E) | Latitiude  (°N) | Altitude  (m) | pH | DO  (mg/L) | T  (℃) | EC  (μs/cm) | TDS  (ppm) | TOC  (mg/L) | COD  (mg/L) | Chl_a  (ug/L) | TN  (mg/L) | TP  (mg/L) | Ammonia  (mg/L) |
| --- | --- | --- | --- | --- | --- | --- | --- | --- | --- | --- | --- | --- | --- | --- |
| S1 | 104.08031 | 29.07582 | 264 | 8.49 | 7.5 | 25.4 | 219 | 110 | 10.6 | 7.44 | 2.06 | 0.576 | 0.097 | 0.596 |
| S2 | 104.28115 | 28.89550 | 247 | 8.41 | 7.1 | 25.3 | 227 | 114 | 10.7 | 7.47 | 2.35 | 0.631 | 0.134 | 0.570 |
| S3 | 104.50683 | 28.77689 | 232 | 8.46 | 6.9 | 26.3 | 222 | 111 | 10.8 | 7.54 | 2.31 | 0.859 | 0.071 | 0.460 |
| S4 | 104.43785 | 28.62364 | 233 | 8.60 | 7.0 | 27.1 | 419 | 210 | 2.80 | 2.08 | 1.55 | 0.048 | 0.004 | 0.226 |
| S5 | 104.60030 | 28.74081 | 235 | 8.63 | 7.5 | 25.6 | 419 | 210 | 2.82 | 2.09 | 1.82 | 0.301 | 0.009 | 0.632 |
| S6 | 104.73152 | 28.77318 | 230 | 8.45 | 6.5 | 26.9 | 371 | 186 | 5.41 | 3.85 | 2.52 | 1.067 | 0.028 | 0.440 |
| S7 | 104.90771 | 28.77257 | 220 | 8.45 | 6.6 | 29.5 | 359 | 180 | 5.40 | 3.84 | 2.82 | 0.357 | 0.028 | 0.677 |
| S8 | 105.05242 | 28.73910 | 211 | 8.86 | 10.03 | 29.8 | 394 | 197 | 5.77 | 4.06 | 2.38 | 0.114 | 0.018 | 0.650 |
| S9 | 105.23241 | 28.74278 | 210 | 8.80 | 8.5 | 27.4 | 366 | 183 | 6.51 | 4.56 | 3.17 | 0.255 | 0.032 | 0.292 |
| S10 | 105.36272 | 28.77044 | 227 | 8.52 | 6.9 | 27.1 | 346 | 173 | 5.6 | 4 | 1.52 | 0.175 | 0.026 | 0.162 |
| S11 | 105.43797 | 28.86612 | 209 | 8.56 | 7.0 | 26.9 | 361 | 181 | 7.7 | 5.4 | 2.60 | 0.586 | 0.029 | 0.137 |
| S12 | 105.56056 | 28.89252 | 183 | 8.58 | 8.2 | 24.3 | 368 | 184 | 7.4 | 5.2 | 2.54 | 0.056 | 0.027 | 0.144 |
| S13 | 105.62629 | 28.86695 | 173 | 8.54 | 7.8 | 26.5 | 362 | 181 | 7.0 | 4.9 | 2.62 | 0.286 | 0.038 | 0.328 |
| S14 | 105.81979 | 28.82931 | 171 | 8.63 | 6.4 | 27.3 | 365 | 183 | 7.4 | 5.2 | 6.98 | 0.119 | 0.032 | 0.165 |
| S15 | 105.91692 | 28.86556 | 172 | 8.57 | 7.2 | 28.3 | 380 | 190 | 9.2 | 6.4 | 4.37 | 0.058 | 0.048 | 0.206 |
| S16 | 105.83103 | 28.96503 | 164 | 8.89 | 8.7 | 27.1 | 371 | 186 | 8.58 | 6.0 | 8.58 | 0.098 | 0.053 | 0.205 |
| S17 | 105.89637 | 29.05068 | 177 | 8.54 | 7.2 | 26.9 | 392 | 196 | 7.59 | 5.31 | 3.63 | 0.846 | 0.044 | 0.099 |
| S18 | 106.09593 | 29.06454 | 158 | 8.60 | 7.3 | 26.2 | 371 | 186 | 8.75 | 6.13 | 3.05 | 0.001 | 0.042 | 0.124 |
| S19 | 106.15982 | 29.21996 | 154 | 8.84 | 7.9 | 26.9 | 375 | 188 | 7.2 | 5.04 | 3.31 | 0.097 | 0.047 | 0.090 |
| S20 | 106.33501 | 29.26061 | 139 | 8.95 | 8.6 | 28.8 | 373 | 187 | 7.93 | 5.55 | 4.81 | 0.006 | 0.056 | 0.102 |
| S21 | 106.42271 | 29.34736 | 138 | 8.50 | 7.0 | 27.1 | 368 | 184 | 8.03 | 5.62 | 3.34 | 0.145 | 0.042 | 0.042 |
| S22 | 104.68510 | 28.68954 | 241 | 9.01 | 10.8 | 31.6 | 416 | 208 | 3.83 | 2.78 | 3.84 | 3.105 | 0.005 | 0.679 |
| S23 | 104.69359 | 28.71192 | 248 | 8.88 | 12.9 | 34.9 | 425 | 213 | 4.04 | 2.92 | 4.67 | 3.070 | 0.001 | 0.488 |
| S24 | 104.68217 | 28.73362 | 247 | 8.46 | 8.6 | 32.7 | 427 | 214 | 4.05 | 2.94 | 3.68 | 3.521 | 0.002 | 0.438 |
| S25 | 104.94463 | 28.63607 | 214 | 8.58 | 6.8 | 34.1 | 641 | 321 | 6.7 | 4.7 | 9.71 | 3.230 | 0.160 | 0.757 |
| S26 | 104.98804 | 28.66635 | 247 | 8.82 | 13.5 | 38.8 | 1023 | 512 | 5.8 | 4.1 | 13.45 | 1.850 | 0.064 | 0.202 |
| S27 | 105.04916 | 28.72476 | 209 | 9.09 | 9.5 | 34.3 | 559 | 280 | 12.5 | 8.8 | 23.95 | 2.558 | 0.075 | 0.362 |
| S28 | 105.37387 | 28.67628 | 231 | 8.20 | 6.8 | 34.5 | 460 | 230 | 5.5 | 3.9 | 2.78 | 1.150 | 0.009 | 0.239 |
| S29 | 105.41651 | 28.72132 | 234 | 8.61 | 7.9 | 33.9 | 476 | 238 | 6.5 | 4.6 | 8.06 | 1.080 | 0.013 | 0.250 |
| S30 | 105.40745 | 28.75793 | 219 | 8.94 | 9.5 | 35.7 | 463 | 232 | 8.5 | 6.0 | 13.21 | 1.649 | 0.032 | 0.199 |
| S31 | 105.34579 | 28.94513 | 194 | 8.76 | 7.0 | 32.3 | 452 | 226 | 9.0 | 6.3 | 11.23 | 1.567 | 0.092 | 0.279 |
| S32 | 105.37600 | 28.90904 | 195 | 8.72 | 6.2 | 33.1 | 457 | 229 | 9.1 | 6.4 | 7.17 | 1.848 | 0.082 | 0.354 |
| S33 | 105.41338 | 28.88483 | 186 | 8.76 | 7.5 | 33.1 | 455 | 228 | 8.8 | 6.1 | 8.61 | 1.991 | 0.081 | 0.725 |
| S34 | 104.81491 | 27.49752 | 1537 | 8.95 | 8.12 | 23.2 | 256 | 128 | 6.73 | 4.71 | 0.57 | 2.038 | 0.021 | 0.296 |
| S35 | 105.03142 | 27.68300 | 1093 | 8.8 | 7.1 | 21.3 | 334 | 167 | 8.44 | 5.91 | 0.73 | 9.188 | 0.046 | 0.223 |
| S36 | 105.58279 | 27.72054 | 569 | 8.79 | 7.7 | 23.7 | 372 | 186 | 6.8 | 4.8 | 1.4 | 5.797 | 0.056 | 0.226 |
| S37 | 105.72451 | 27.70660 | 492 | 8.75 | 6.8 | 23.3 | 375 | 188 | 6.84 | 4.79 | 1.16 | 6.001 | 0.032 | 0.174 |
| S38 | 105.93263 | 27.72839 | 438 | 8.67 | 7.2 | 24 | 375 | 188 | 5.98 | 4.19 | 1.37 | 5.264 | 0.018 | 0.143 |
| S39 | 106.37023 | 27.85319 | 370 | 8.79 | 8.9 | 25.2 | 384 | 192 | 5.86 | 4.12 | 3.05 | 4.652 | 0.016 | 0.072 |
| S40 | 106.32899 | 28.01633 | 343 | 8.67 | 5.3 | 25.7 | 402 | 201 | 6.28 | 4.4 | 3.94 | 5.471 | 0.026 | 0.050 |
| S41 | 106.16642 | 28.15341 | 291 | 8.09 | 4.5 | 26.1 | 432 | 216 | 4.78 | 3.44 | 1.90 | 3.579 | 0.024 | 0.076 |
| S42 | 106.04566 | 28.16309 | 266 | 8.53 | 6.8 | 26.1 | 406 | 203 | 6.41 | 4.49 | 6.34 | 3.648 | 0.021 | 0.112 |
| S43 | 106.00695 | 28.26293 | 259 | 8.52 | 7.4 | 30.8 | 528 | 264 | 6.40 | 4.48 | 2.44 | 3.350 | 0.020 | 0.187 |
| S44 | 105.94503 | 28.47032 | 212 | 8.57 | 6.9 | 28.7 | 409 | 205 | 6.70 | 4.69 | 4.71 | 4.628 | 0.010 | 0.051 |
| S45 | 105.68667 | 28.53253 | 202 | 8.68 | 8.1 | 28.4 | 397 | 199 | 6.18 | 4.33 | 6.38 | 4.116 | 0.030 | 0.240 |
| S46 | 105.73745 | 28.61214 | 186 | 8.58 | 6.8 | 27.4 | 400 | 200 | 6.48 | 4.54 | 4.57 | 6.542 | 0.023 | 0.021 |
| S47 | 105.7271 | 28.71312 | 178 | 8.65 | 6.6 | 27.1 | 409 | 205 | 7.34 | 5.14 | 8.72 | 3.116 | 0.029 | 0.045 |
| S48 | 105.7878 | 28.78364 | 173 | 8.58 | 6.7 | 31.1 | 384 | 192 | 9.63 | 6.74 | 22.51 | 2.853 | 0.037 | 0.669 |
